# Cortisol profiles and clinical severity in *MECP2* duplication syndrome

**DOI:** 10.1186/s11689-020-09322-5

**Published:** 2020-07-22

**Authors:** Sarika U. Peters, Cary Fu, Jeffrey L. Neul, Douglas A. Granger

**Affiliations:** 1 Vanderbilt University Medical Center, Nashville, USA; 2 Deparment of Pediatrics, Vanderbilt University Medical Center, Vanderbilt Kennedy Center, PMB 74, 230 Appleton Place, Nashville, TN 37203-5721 USA; 3 University of California, Irvine, and Johns Hopkins University, Baltimore, USA

## Abstract

**Background:**

*MECP2* duplication syndrome (MDS) is a rare X-linked genomic disorder primarily affecting males which is caused by interstitial chromosomal duplications at Xq28 encompassing the *MECP2* gene. Core clinical features of MDS include choreiform movements, progressive spasticity, recurrent respiratory infections, developmental delays in the first 6 months of life, hypotonia, vasomotor disturbances, constipation, drooling, and bruxism. Prior studies suggest that HPA axis activity may be altered in MDS and measures of HPA axis activity may offer insight into disease severity.

**Methods:**

To ascertain whether cortisol profiles are a potential biomarker of clinical severity, diurnal profiles of cortisol and the cortisol awakening response were examined from saliva samples in 31 participants with MDS (ages 2–24 years), and 27 of these samples were usable. Documentation of a positive diagnostic test for *MECP2* duplication was required for entry into the study. Samples were collected on each of two consecutive weekdays at four time points during the day: immediately after waking, 30 min after waking, between 3 and 4 PM, and in the evening before bedtime. Correlations with duplication size, clinical severity, sleep problems, and behavior were also examined.

**Results:**

Results revealed that a majority of participants with MDS exhibit a declining cortisol awakening response (*n* = 17). A declining CAR was significantly associated with increased clinical severity scores (*r* = − .508; *p* = .03), larger duplication size, waking later, and an increased number of hospitalizations for infections.

**Conclusions:**

Future mechanistic studies will have to determine whether the declining CAR in MDS is attributable to problems with “flip-flop switching” of regional brain activation (involving the suprachiasmatic nucleus and the hippocampus, and the HPA axis) that is responsible for the switch from reduced to increased adrenal sensitivity. Taken together, results suggest the possibility that cortisol profiles could potentially be a biomarker of clinical severity and utilized for the purposes of patient stratification for future clinical trials in MDS.

## Introduction

*MECP2* duplication syndrome (MDS) is a rare X-linked genomic disorder primarily affecting males which is caused by interstitial chromosomal duplications at Xq28 encompassing the *MECP2* gene [1]. This gene encodes methyl CpG binding protein 2 (MeCP2), a regulator of neuronal gene transcription that is required for normal brain maturation [2, 3]. Loss of MeCP2 function is the primary cause of Rett syndrome (RTT), a distinct neurogenetic disorder primarily affecting females exhibiting some symptom overlap with MDS. Our prior studies reveal considerable variability in severity of many symptom domains within MDS including ambulation, hand function, non-verbal communication, and susceptibility to infection [3]. The core clinical features of MDS include choreiform movements, progressive spasticity, and recurrent respiratory infections [1, 2] as well as developmental delays in the first 6 months of life, hypotonia, vasomotor disturbances, constipation, drooling, and bruxism [3]. We also discovered duplication size and specific gene content play a role in clinical severity [3]. The recent report of phenotypic reversal after antisense oligonucleotide treatment in an animal model of MDS [4] suggests disease modification may be possible and candidate therapeutics are already in development. The eventual clinical evaluation of these therapeutics will require well-defined, quantifiable measures to assess outcomes. As such, it is essential to identify these disease biomarkers in preparation for upcoming clinical trials.

The high risk of recurrent lower respiratory tract infections in MDS is believed due to immune dysregulation and chronic inflammation [5]. The hypothalamus-pituitary-adrenal (HPA) axis has an important role in immune regulation and is dysfunctional in the autoimmune disorders systemic lupus erythematosus and Sjögren’s syndrome [6]; both of which exhibit *MECP2* overexpression [7, 8]. Studies in other populations [9–11] further suggest that HPA axis hypoactivity may contribute to increased frequency and/or severity of infections and systemic inflammation. Production of CRH by the hypothalamus is central to HPA axis function, causing the release of adrenocorticotropic hormone from the pituitary gland, which in turn stimulates the release of cortisol from the adrenal glands. Studies of RTT and MDS in animal models demonstrate direct regulation of *Crh* gene expression by MeCP2 [12, 13]. These observations suggest that HPA axis activity may be altered in MDS and measures of HPA axis activity may offer insight into disease severity.

Levels of cortisol are high on waking, increase dramatically from waking to 30 min post-waking, decline rapidly before midday, and then gradually decline across the PM hours. Low cortisol levels in the morning are noted in rheumatoid arthritis, lupus, and Sjögren’s syndrome [14, 15]. The cortisol awakening response (CAR) refers to the rapid increase in cortisol secretion within the first 30 min of wakening, signifying a transition of the suprachiasmatic nucleus (SCN) from an inhibitory to an excitatory state. Research studies consistently show that impaired hippocampal function (including reduced volume) contributes to an attenuated CAR [16, 17]. An attenuated CAR (i.e., inverse-declining slope) has been associated with autoimmune disorders, depression, anxiety, and cognitive impairment [18, 19].

Our prior pilot study [20] examined diurnal patterns in salivary cortisol in four males with *MDS* who had regression and four males without regression. Individuals who had experienced regression exhibited flatter cortisol production through the day and a negative slope (i.e., declining levels of cortisol from awakening through the day to bedtime). In contrast, individuals with *MECP2* duplication syndrome who had not experienced regression showed more typical patterns of higher cortisol levels in the morning with linear decreases throughout the day. To expand on this initial work, and to begin to ascertain whether cortisol profiles are a potential biomarker of clinical severity, we examine diurnal profiles of cortisol and the CAR in participants with MDS and examine relationships with duplication size, clinical severity, sleep problems, and behavior.

## Methods

### Participants

This cohort of patients is part of a broader longitudinal study of MDS related to markers of disease progression (*n* = 38 to date). A subset of these participants are also part of a longitudinal natural history study of MDS [3], Rett syndrome, and other Rett-related disorders. Although 31 participants, ranging between the ages of 2 and 24 years, have completed and analyzed saliva samples from a baseline enrollment visit to date, 27 of these are presented here. The additional four samples were excluded due to insufficient quantities of saliva for analysis, and/or a missing sample. Documentation of a positive diagnostic test for *MECP2* duplication was required for entry into the study. Most participants had their duplications detected via Array Comparative Genomic Hybridization (Array-CGH), while others (mostly older participants) had Multiplex-Ligation-dependent Probe Amplification (MLPA). Participants in this study are evaluated on a yearly basis. All participants had remote gathering of parental reported data, and a subset (*n* = 20) have also contributed in-person clinician and parent-reported data. Due to the limited number of participants thus far assessed for longitudinal follow-up, only baseline data is being reported at this time. The families of all participants provided written informed consent, and all procedures performed in the studies were done in accordance with the ethical standards of the institutional research committee.

### Measures

#### Clinical Severity Scale (CSS)—clinician rating

The CSS was developed for use in RTT and has been used to assess almost 2000 children, adolescents, and adults with RTT and related disorders who have been enrolled in the natural history study [21]. It is a composite score based on thirteen individual ordinal categories measuring common clinical features during an in-person exam (e.g., independent sitting, hand use, scoliosis, language, seizures, autonomic symptoms, onset of stereotypies, regression, and head growth). Individual item scores range from 0 to 4 or 0 to 5 with 0 representing the least severe and 4 or 5 representing increasing severity [22]. Lower total scores represent milder severity (see supplementary files).

#### Motor Behavioral Assessment Scale (MBA)—clinician rating

The MBA has also been used to assess almost 2000 children, adolescents, and adults with RTT and related disorders who have been enrolled in the natural history study [21]. The current version of this scale consists of 34 items across three subscales: behavior/social (irritability, aggression, poor eye gaze, sustained interest, etc.), orofacial (bruxism, mouthing objects or hands, biting self, breath holding, hyperventilation, etc.), and motor (bradykinesia, dystonia, ataxia, chorea, etc.), the scores of which are based on current functioning. These are scored during a clinical interview and in-person exam by a specialist once per year. Items are captured on a 5-point Likert scale. Lower total scores indicate milder disease severity (see supplementary files).

#### Aberrant Behavior Checklist (ABC)

The ABC is a caregiver-rated behavior rating scale that measures irritability, stereotypy, social withdrawal, and hyperactivity and has been used in our previous studies of MDS [23].

#### Additional clinical features (seizures, anxiety, sleep problems, hospitalizations, etc.)

Salient clinical features were quantified via parent report and clinician observation according to a scale (none, occasional, frequent, very frequent, constant). They were asked about symptoms that had occurred within the past year. For the purposes of this study, these were recoded to determine the presence or absence of these features.

### Determination of salivary cortisol

Given the functional level of participants and potential risks for choking, saliva was collected at home by parents/caregivers using a 1 × 12.5 cm absorbent swab specifically designed for use with children (Salivabio, Carlsbad, CA). Samples were collected on each of two consecutive weekdays at four time points during the day: immediately after waking, 30 min after waking, between 3 and 4 PM, and in the evening before bedtime. After collection, samples were immediately frozen in the families’ home freezers and remained frozen during transport to the University. On the day of assay, samples were thawed and centrifuged to remove mucins. Samples were assayed in duplicate for cortisol (ug/dL) using commercially available immunoassay protocols specifically designed for use with saliva without modification to the manufacturer’s recommended protocol (Salimetrics, Carlsbad, CA). The test volume was 25 μL, lower limit of sensitivity 0.007 μg/dL, range of calibrators .007–3.0 ug/dL, and, on average, the inter- and intra-assay coefficients of variation were less than 15% and 10%. Cortisol values used in the statistical analyses were averaged across duplicates within day and collection time point in line with standard practice [24–26]. Quality control was executed, for sample collection time point adherence specifically, by excluding any samples that were not collected during the specified time points, as was checked by coordinator verification of time stamps, and as noted by the samples excluded from the overall analyses.

### Analysis

Derived cortisol variables were calculated including the cortisol awakening response (CAR), the area under the curve with respect to ground (AUCG), and the area under the curve with respect to increase (AUCI). The CAR represents the increase in cortisol from waking to 30 min post-waking and was calculated as the difference score between samples 1 and 2. Importantly, the CAR was measured in accordance with consensus guidelines [27]. AUCG represents the difference between the single measurements from each other (i.e., the change over time) and the distance of these measurements from the ground/zero. AUCI is calculated from the formula for AUCG, since it is identical to AUCG except for the removal of the area between ground and the first measure (baseline) for all time points as is specified in prior research studies [28].

Pearson correlation coefficients were calculated to examine the relationships between cortisol variables and measures of clinical severity (CSS and MBA). Participants were then divided into groups according to CAR value (expected morning rise in the first 30 min post-awakening vs. decline in cortisol levels in the first 30 min post-awakening). Using ANOVAs, differences were examined for duplication size, and for behavior using the ABC. For categorical variables including the presence vs. absence of seizures, hospitalizations for infections, and sleep problems (i.e., problems falling asleep, night-waking, difficulties waking up in the morning), the likelihood ratio test (a form of chi-square recommended for small samples) was performed to examine any relationship between a positive vs. negative CAR and the presence vs. absence of these difficulties.

## Results

### Demographics

The mean age of participants at evaluation was 8.18 years (SD = 5.88 years). As is per the convention in MDS, the majority of participants were male (*n* = 25) and two were female (Table 1). All participants in this study lived in the home; 58% of mothers and 52% of fathers had a bachelor’s or advanced degree.

**Table 1:** Demographics, CAR profiles, clinical severity, and duplication size

Participant	Age (years)	Gender	AUCI	AUCG	CAR positive or declining	CSS	MBA	Gene duplication size (BP)	Hospitalizations due to infections
1	11	M	− 104.26	108.97	Declining	N/A	N/A	1267181	
2	10	M	− 638.39	253.43	Declining	26	48	1132418	
3	12	M	− 127.29	106.80	Declining	19	36	452833	
4	8	M	− 12.20	95.54	Increasing	11	28	453484	
5	5	F	− 7.16	166.19	Increasing day 1 Declining day 2	N/A	N/A	N/A	
6	4	M	− 1.30	46.36	Declining	7	11	253731	X
7	3	M	− 360.15	176.88	Declining	12	17	1615017	
8	3	M	− 18.96	178.68	Declining	9	11	698611	X
9	4	M	− 20.43	377.28	Increasing	16	34	14461013	
10	9	M	− 187.88	− 3.01	Declining	17	33	663918	
11	8	M	− 60.75	− 1.08	Declining	17	28	698143	
12	9	M	− 13.38	− .90	Declining	10	22	443304	
13	11	M	− 103.89	− .71	Declining	11	38	210970	
14	9	M	38.85	1.83	Increasing	10	26	663047	
15	6	F	176.14	.94	Increasing	14	30	6007098	
16	14	M	− 92.69	1.30	Increasing	20	32	N/A	
17	2	M	− 260.16	− 1.32	Declining	26	49	3062065	X
18	5	M	− 375.63	− 3.42	Declining	11	20	866029	
19	2	M	− 94.07	− .85	Declining	11	31	382168	
20	9	M	2.37	− .15	Declining	N/A	N/A	469000	
21	24	M	− 263.79	− 3.75	Declining	N/A	N/A	N/A	X
22	6	M	− 1.62	3.36	Increasing	N/A	N/A	279323	
23	2	M	− 57.73	4.19	Increasing	N/A	N/A	2644599	
24	21	M	19.06	.83	Increasing	10	20	476834	
25	19	M	− 115.97	− .95	Declining	N/A	N/A	N/A	X
26	3	M	− 430.86	− 3.06	Declining	13	25	254752	X
27	2	M	204.97	8.54	Increasing	11	22	224380	

*CAR* cortisol awakening response, *AUCI* area under the curve with respect to increase, *AUCG* area under the curve with respect to ground, *BP* base pairs

### Diurnal cortisol profiles

**Fig. 1:**
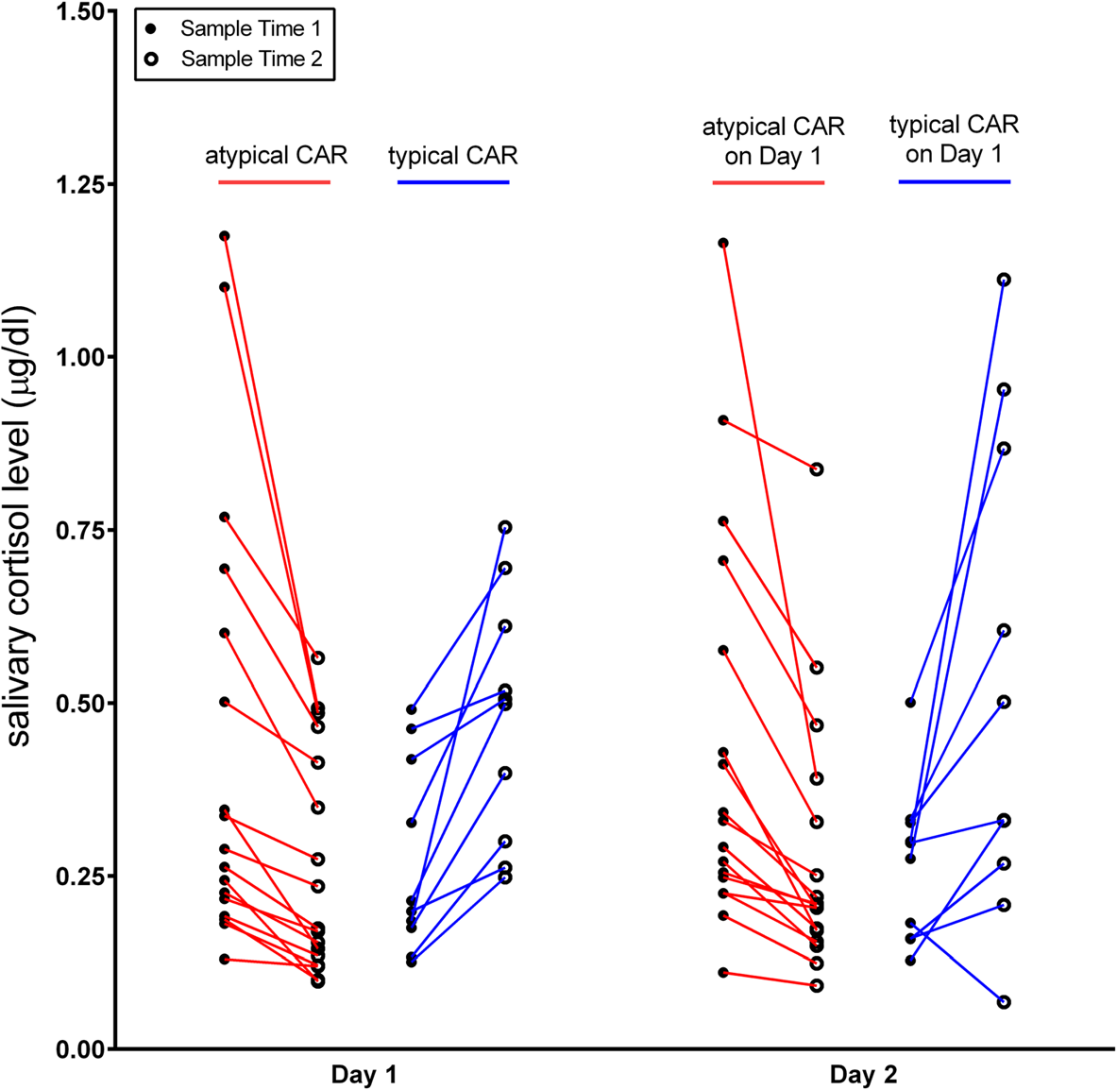
Individual cortisol patterns across day 1 and day 2 for each participant

**Fig. 2:**
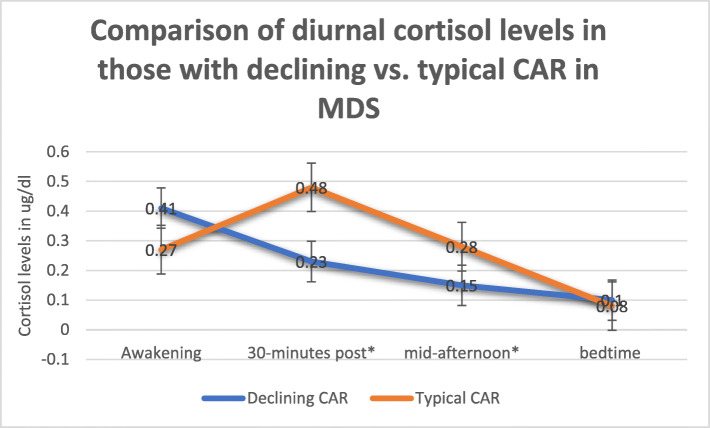
Comparison of diurnal cortisol levels for those with declining vs. typical CAR responses in MDS

Results revealed that there were no correlations with chronological age for average cortisol levels, AUCI, or AUCG throughout the day, or for any of the individual time points. Of the 27 participants, 17 had an absent/declining CAR on both days of cortisol collection, meaning that their cortisol level dropped from the time that they first woke to 30 min post-waking (see Table 1). Nine participants had a more typical CAR on both days of collection, with cortisol levels rising from waking to 30 min post-waking (see Table 1). One child had a more typical CAR on the first day of collection, but a declining profile on the second day of collection. Because of this mismatch of her CAR pattern across collection days, she was excluded from subsequent analyses when examining correlations with clinical parameters (see Supplementary Table 1 for raw values for each participant). Figure 1 shows the individual CAR responses divided into groups according to those who had a declining CAR vs. those who had a more typical profile. Figure 1 also depicts the pattern of a child who had an increasing profile on day 1, but a decreasing profile on day 2. Figure 2 shows the mean cortisol levels for each time point across both groups and also demonstrates that the participants who had a more typical CAR had significantly higher cortisol levels at the 2nd (*F* = 15.87; *p* = .001) and 3rd (*F* = 3.84; *p* = .05) time points. Although the subset of participants with a declining CAR had a slightly higher awakening level of cortisol as compared to those with a more typical profile, this was not statistically significant (Fig. 2). There were no significant differences in age between those who had a declining CAR and those who had a typical profile. Formal statistics were not done to examine differences by gender given the imbalance across groups.

### Correlations with clinical parameters

In examining correlations between the CSS and the MBA and cortisol parameters, a declining CAR was significantly associated with increased clinical severity scores (CSS) (*r* = − .508; *p* = .03) (Table 1). A more negative area under the curve with respect to increase (AUCI) was also associated with an increased CSS (*r* = − .481; *p* = .05). Correlations with the MBA were in the same direction but were not statistically significant. There were no significant associations between AUCG and either the CSS or the MBA.

Data regarding duplication size and differences in CAR profiles were also examined (Table 1). Information on specific breakpoints was available for 23 of 27 participants. Duplication sizes ranged between 210,970 and 14,461,013 bp. Those with a declining CAR profile were significantly more likely to have a larger duplication size [*F* = 4.14; *p* < .05; *M* = 831342.667 (SD = 737433) vs. typical profile *M* = 3561493.57 (SD = 5233440)]. The results of chi-squared tests showed that participants whose parents reported trouble waking over the past year had a declining CAR (*X*^2^ = 4.10; *p* = .04). There were no significant differences noted for night-waking, sleeping during the day, or problems falling asleep at night. Only those with a negative CAR were hospitalized due to infections over the past year (*X*^2^ = 3.77; *p* = .05) (Table 1). Other findings were not significant.

Table 2 shows the results of CAR profiles and the ABC. No statistically significant results were noted, although there was a trend such that those with a negative CAR profile had higher scores on the Lethargy/Withdrawal subscale of the ABC.

**Table 2:** Mean scores on the Aberrant Behavior Checklist by CAR pattern

	Irritability	Lethargy/social withdrawal	Stereotypy	Hyperactivity
Negative CAR	3.36	8.86	5.50	10.00
Positive CAR	3.50	4.30	5.70	6.60
F and p values	*F* = .005; *p* = NS	*F* = 2.42; *p* = .10	*F* = .011; *p* = NS	*F* = .738; *p* = NS

## Discussion

This study represents the first detailed examination of diurnal cortisol profiles in participants with MDS and how these profiles correspond to clinical severity, gene duplication size, sleep problems, and behavior. These findings extend our previous pilot study [20] by demonstrating that a significant proportion of those with MDS (63% of this sample) have an atypical diurnal cortisol profile marked by an attenuated CAR (i.e., no rise in cortisol within the first 30 min of awakening). Rather, this subgroup of participants started with a statistically equivalent level of first morning cortisol (slightly higher numerically), but then their levels declined throughout the rest of the day. In contrast, the other subgroup of participants exhibited a more typical pattern. Population studies of 18,698 individuals across the age range show that this pattern of an absent/declining CAR is extremely rare [29]. An attenuated CAR has been noted especially in clinical populations with impaired hippocampal functioning [16]. It has been hypothesized that the hippocampus plays a role in regulation of the CAR prior to awakening [17]. More specifically, it is hypothesized that hippocampal activation during the pre-awakening period is implicated in regulation of pre-awakening cortisol secretion. The hippocampus is active during REM sleep and becomes inhibited during non-REM sleep and awakening. Typically, there is reduced adrenal sensitivity to ACTH in the pre-awakening period, and this is mediated by the suprachiasmatic nucleus (SCN) [16]. In the subgroup of participants with MDS who have a declining CAR, there is the possibility that there is less pre-awakening reduced adrenal sensitivity, resulting in a higher level of cortisol for sample 1. Interestingly, the MeCP2 protein is highly expressed in the SCN [30]. Although studies have not been done in MDS animal models, studies in RTT animal models find that RTT mice wake later and have a highly fragmented sleeping pattern due to deficits in the circadian timing system (which is controlled by the SCN) [30]. Sleep problems are commonly reported in clinical studies in RTT [31, 32], but have been researched to a lesser extent in MDS. In RTT, 79–85% of caregivers reported their children experienced at least one sleep problem, including frequent nighttime waking, screaming spells and/or laughing at night, or daytime sleepiness [32]. In this study, it is notable that the subgroup of participants with MDS who had a declining CAR tended to wake later, although there were no differences noted in other aspects of sleep. Future mechanistic studies will have to determine whether the declining CAR in MDS is attributable to problems with “flip-flop switching” of regional brain activation (involving the SCN and the hippocampus, and the HPA axis) that is responsible for the switch from reduced to increased adrenal sensitivity.

An attenuated CAR, as well as a negative AUCI, was associated with greater clinical severity as measured by the clinical severity scale (CSS). The CSS measures a variety of salient clinical features in MDS, and these findings suggest the possibility, pending longitudinal studies, that an attenuated CAR could be a marker of disease severity and progression. This also mirrors findings in other disorders (e.g., depression, anxiety, cognitive impairment, cardiac disease) [33–35]. Given that a declining CAR is also associated with having a larger duplication, this finding also adds to our previous study [3] suggesting that those with larger duplication sizes exhibit greater clinical severity. Data regarding potential contributions of individual genes within the breakpoint interval has been reported in our previous study [3] and was not examined within this cohort given the relatively smaller sample size, especially within specific groups once they were separated by CAR profiles.

Recurrent infections are part of a core phenotype in MDS [1], and the present findings suggest that those with more hospitalizations for infections were more likely to have a declining CAR, although this finding should be replicated in larger samples. MDS results from non-recurrent duplications of Xq28, and the shared region of overlap among affected individuals also spans the *IRAK1* (interleukin-1 receptor-associated kinase 1). *IRAK1* is important for the activation and regulation of innate and adaptive immunity. A previous study suggests that independent of aberrant expression of IRAK1, there is a primary role for the overexpression of MeCP2 in mediating the immune deficiencies seen in *MECP2* duplication syndrome [5]. The abnormalities confer a selective alteration in the capacity of helper (CD4+) T cells (T_H_1) to develop into the subset that normally produces the cytokine interferon-γ (IFN**-**γ) [5]. The degree to which CAR profiles and cortisol parameters correspond to concentrations of Th1 helper cells and specific immune markers should therefore be examined in future studies.

There were no significant differences in CAR profiles for any behavioral parameters as measured on the ABC, although there was a trend level finding that those with a negative CAR exhibited increased social withdrawal. Social withdrawal has been associated with “sickness” behavior, increased susceptibility to infections, and persistent inflammation [36] in other studies. As such, these behavioral patterns should continue to be examined in larger cohorts of individuals with MDS as well as longitudinally.

There are some limitations to the present study that are important to note. It is possible that parents, in spite of determining times of awakening with the help of video monitors, failed to correctly report/determine their child’s time of awakening and this could have impacted CAR profiles. This could certainly have been the case for the child who exhibited an increasing profile on day 1 and a declining profile on day 2 of collection. Specifically, other studies have found that delaying the collection of sample 1 after awakening by more than 15 min results in false-high estimates of sample 1 and false-low estimates of the CAR [37]. Future studies in this cohort should therefore utilize polysomnography and sleep actigraphy with electronic devices to further verify times of awakening and to better insure the most accurate sample collection times post-awakening. In addition, these data are cross-sectional, and it will be important to examine how CAR profiles change longitudinally in this population especially if there is an onset of regression [38] and/or seizures. Although duplication size was examined, mRNA levels were not examined to gauge the levels of MeCP2 protein that were being expressed and this could reveal some important insights into clinical severity and cortisol profiles. In addition, it will be important in future studies to use standardized, quantifiable measures of sleep and sleep quality (e.g., actigraphy, the Child Sleep Habits questionnaire) so that additional insights can be gleaned in order to place these results within context.

## Conclusion

To summarize, this is the first study in MDS to examine the CAR and how this corresponds to clinical severity and heterogeneity in clinical presentations. Considering the continued progress toward clinical trials in MDS, the possibility that cortisol profiles could potentially be a biomarker of clinical severity and utilized for the purposes of patient stratification should continue to be examined especially given that these measurements can be done remotely and are non-invasive. These data are also invaluable in the further development of patient assessment tools that are standardized, dynamic, valid, and reliable to accurately measure outcomes.

## Supplementary information

**Additional file 1:.** Supplementary Table 1: Raw Cortisol Values for Day 1 and Day 2.

## Data Availability

The datasets used and/or analyzed during the current study are available from the corresponding author on reasonable request
