# Supplementary material for: Cortisol profiles and clinical severity in MECP2 duplication syndrome
Source: J Neurodev Disord. 2020 Jul 22;12:19. doi: 10.1186/s11689-020-09322-5 (PMC7376951; doi:10.1186/s11689-020-09322-5)
Supplement: Supplementary file 1 — Additional file 1:. Supplementary Table 1: Raw Cortisol Values for Day 1 and Day 2. [file 11689_2020_9322_MOESM1_ESM.docx]

Supplementary Table 1: Raw Cortisol Values for Day 1 and Day 2

| Participant | Cortisol Day 1 Time 1 | Cortisol Day 1 Time 2 | Cortisol Day 2 Time 1 | Cortisol Day 2 Time 2 |
| --- | --- | --- | --- | --- |
| 1 | .263 | .174 | .412 | .204 |
| 2 | 1.101 | .486 | .909 | .838 |
| 3 | .289 | .235 | .226 | .155 |
| 4 | .133 | .300 | .128 | .330 |
| 5* | .214 | .499 | .182 | .068 |
| 6 | .187 | .100 | .342 | .220 |
| 7 | 1.175 | .493 | 1.165 | .391 |
| 8 | .244 | .098 | .225 | .204 |
| 9 | .491 | .695 | .501 | .868 |
| 10 | .346 | .145 | .255 | .208 |
| 11 | .226 | .154 | .248 | .210 |
| 12 | .181 | .121 | .292 | .174 |
| 13 | .217 | .170 | .111 | .092 |
| 14 | .126 | .248 | .160 | .208 |
| 15 | .199 | .262 | .298 | .331 |
| 16 | .419 | .506 | .275 | .953 |
| 17 | .502 | .414 | .429 | .171 |
| 18 | .694 | .466 | .706 | .468 |
| 19 | .192 | .135 | .193 | .124 |
| 20 | .130 | .120 | .271 | .149 |
| 21 | .601 | .349 | .576 | .328 |
| 22 | .175 | .399 | .159 | .268 |
| 23 | .327 | .611 | .332 | .605 |
| 24 | .463 | .518 | .327 | .502 |
| 25 | .337 | .274 | .330 | .251 |
| 26 | .769 | .565 | .763 | .551 |
| 27 | .185 | .754 | .300 | 1.112 |

* Denotes the participant who exhibited an increasing CAR profile on day 1, but a decreasing profile on day 2. Her data was excluded from further analyses examining correlations with clinical parameters.
